# Artificial Intelligence Chatbots in Peritoneal Dialysis Education: A Cross-Sectional Comparative Study of Quality, Readability, and Reliability

**DOI:** 10.3390/jcm15020692

**Published:** 2026-01-15

**Authors:** Engin Onan, İlter Bozaci, Yelda Deligoz Bildaci, Sevinc Puren Yucel Karakaya, Ruya Kozanoglu, Rumeyza Kazancioglu

**Affiliations:** 1 Department of Nephrology, Adana Dr. Turgut Noyan Training and Research Hospital, Faculty of Medicine, Baskent University, Adana 01120, Turkey; rusancak@hotmail.com; 2 Department of Nephrology, Antalya City Training and Research Hospital, Muratpaşa 07100, Turkey; ilterbozaci@gmail.com; 3 Department of Nephrology, Faculty of Medicine, Dokuz Eylul University, Izmir 35340, Turkey; yeldadeligoz@gmail.com; 4 Department of Biostatistics, Faculty of Medicine, Cukurova University, Adana 01330, Turkey; sevincpurenyucel@gmail.com; 5 Department of Nephrology, Faculty of Medicine, Bezmialem Vakif University, Istanbul 34093, Turkey; rumeyza@hotmail.com

**Keywords:** peritoneal dialysis, artificial intelligence chatbots, readability and reliability, patient education

## Abstract

**Background:** Peritoneal dialysis (PD) remains underutilized worldwide, partly due to limited patient education, misconceptions, and barriers to accessing reliable health information. Artificial intelligence (AI)-based chatbots have emerged as promising tools for improving health literacy, supporting shared decision-making, and enhancing patient engagement. However, concerns regarding content quality, reliability, and readability persist, and no study to date has systematically evaluated AI-generated content in the context of PD. Therefore, this study aimed to systematically evaluate the quality, reliability, and readability of AI-generated educational content on peritoneal dialysis using multiple large language model-based chatbots. **Methods:** A total of 45 frequently asked questions about PD were developed by nephrology experts and categorized into three domains: general information (*n* = 15), technical and clinical issues (*n* = 21), and myths/misconceptions (*n* = 9). Three AI-based chatbots, Gemini Pro 2.5, ChatGPT-5, and LLaMA Maverick 4, were prompted to generate responses to all questions. Each response was independently evaluated by two blinded reviewers for textual characteristics, readability using the Flesch Reading Ease Score (FRES) and Flesch-Kincaid Grade Level (FKGL), and content quality/reliability using the Ensuring Quality Information for Patients (EQIP) tool and the Modified DISCERN instrument. **Results:** Across all domains, significant differences were observed among the chatbots. Gemini Pro 2.5 achieved higher Flesch Reading Ease (FRES) scores (32.6 ± 10.5) compared with ChatGPT-5 (24.2 ± 11.7) and LLaMA Maverick 4 (16.2 ± 7.5; *p* < 0.001), as well as higher EQIP scores (75.4% vs. 59.4% and 61.5%, respectively; *p* < 0.001) and Modified DISCERN scores (4.0 [4.0–4.5] vs. 3.0 [3.0–3.5] and 3.0 [2.5–3.5]; *p* < 0.001). ChatGPT-5 demonstrated intermediate performance, while LLaMA Maverick 4 showed lower scores across evaluated metrics. **Conclusions:** These findings demonstrate differences among AI-based chatbots in readability, content quality, and reliability when responding to identical peritoneal dialysis–related questions. While AI chatbots may support health literacy and complement clinical decision-making, their outputs should be interpreted with caution and under appropriate clinical oversight. Future research should focus on multilingual, multicenter, and outcome-based studies to ensure the safe integration of AI into PD patient education.

## 1. Introduction

PD remains an underutilized modality for the management of kidney failure despite its advantages in terms of cost-effectiveness, patient autonomy, and quality of life [1]. Multiple studies have highlighted that limited patient education, misunderstandings, and barriers to accessing reliable health information significantly contribute to the low adoption rates of PD worldwide [2,3]. Patients frequently encounter difficulties in understanding complex medical terminology, evaluating the reliability of online health information, and distinguishing evidence-based recommendations from misinformation. These factors may negatively influence decision-making, adherence to therapy, and ultimately, clinical outcomes.

In parallel, the rapid development of AI has introduced novel tools that can potentially address some of these challenges. AI-based chatbots, powered by large language models, are increasingly being integrated into various healthcare settings to enhance health literacy, improve patient engagement, and facilitate shared decision-making between patients and clinicians [4]. Previous research in fields such as oncology, hepatology, and cardiovascular medicine has shown that AI-driven chatbots can deliver educational content with reasonable accuracy and user satisfaction [5,6,7]. Moreover, large language models have demonstrated high accuracy and clinical acceptability when responding to physician-generated questions across multiple medical specialties, as highlighted in a recent study evaluating ChatGPT-5 responses to 284 clinical queries, which reported a median accuracy score of 5.5 out of 6 and a completeness score of 3.0 out of 3 [8,9]. Similarly, studies in chronic kidney disease (CKD) have shown that AI-driven chatbots can provide accurate and accessible information, with performance improving across successive versions of ChatGPT-5 Bard AI, and Bing AI [10]. However, these studies also identified limitations such as occasional misleading responses, lack of references, and the need for collaboration between clinicians and AI developers to ensure guideline-based, reliable patient education.

Peritoneal dialysis represents a unique clinical context in which accurate, comprehensible, and accessible patient education materials are essential for informed decision-making and long-term adherence. However, no study to date has systematically evaluated the quality, reliability, and readability of AI-generated content specific to PD. Moreover, variations in model performance, regional access limitations, and the lack of standardized assessment tools pose additional challenges to the safe integration of AI into clinical practice.

Against this background, we hypothesized that AI-based chatbots would differ in content quality, readability, and reliability, with potential implications for patient education in PD. Therefore, this study aimed to provide a comprehensive and multidimensional evaluation of three widely used AI-based chatbots for PD patient education.

## 2. Materials and Methods

Three large language model (LLM) chatbots, ChatGPT-5 (OpenAI), Gemini Pro 2.5 (Google), and Meta LLaMA Maverick 4 (Meta), were evaluated for their responses to frequently asked questions about PD. At the time of the study, these were the AI chatbots that were publicly accessible and available for evaluation.

The question set was developed by nephrology experts to address the most common patient concerns and misunderstandings about peritoneal dialysis (PD). This cross-sectional study was conducted between August 2025 and September 2025, using the most recent publicly available versions of each chatbot. A total of 45 questions were included, categorized into three main domains: General Information (*n* = 15), Technical and Clinical Concerns (*n* = 21), and Myths and misconceptions (*n* = 9). The full list of questions in each category is presented in Table 1. The questions were selected through a combination of online searches (Google) to identify the most frequently asked patient queries regarding PD and the authors’ clinical expertise in managing PD patients, ensuring coverage of both routine clinical inquiries and commonly misunderstood topics. The final list of questions is presented in Table 1 under the specified categories.

The question set was developed by nephrology experts based on their clinical experience in peritoneal dialysis care and informed by commonly encountered patient concerns in routine practice. To further ensure relevance, frequently asked questions were identified through structured online searches (Google) reflecting common patient inquiries regarding PD. Patients were not directly involved in the question development process. This approach was chosen to ensure clinical accuracy and comprehensive coverage of both routine and complex aspects of PD education, while patient involvement is planned as a key component of future studies. Although patients were not directly involved in the development of the question set, the selected questions were deliberately designed to reflect real-life decision-making scenarios commonly discussed during peritoneal dialysis counseling, including employment, caregiving responsibilities, travel, body image concerns, home environment requirements, and social participation. These domains represent key preference-sensitive factors known to influence modality choice and long-term adherence in PD patients.

All questions were submitted to each chatbot using identical wording to ensure standardization. Interactions were conducted in isolated chat windows equivalent to an ‘incognito’ session, with each question asked in a separate, new session without prior prompts or conversational history. No sequential questioning was performed, thereby preventing any carryover effects or contextual memory from influencing subsequent responses. This approach ensured that all responses were generated independently under identical, context-free conditions. Responses were obtained in unprocessed text format without additional prompts and recorded in a “Question–Model–Answer” format using Microsoft Word. In total, 135 responses (45 per model) were collected, and all transcripts were independently cross-verified by a second researcher for accuracy. All questions were presented to the chatbots in English to ensure linguistic consistency and facilitate standardized evaluation. Responses were generated using paid versions of each chatbot to ensure access to the most advanced model capabilities available at the time of evaluation.

Readability was assessed using two validated metrics: the Flesch Reading Ease Score (FRES) and the Flesch-Kincaid Grade Level (FKGL). The FRES ranges from 0 to 100, with higher scores reflecting easier readability, while the FKGL indicates the U.S. school grade level required for comprehension. Both metrics were computed automatically using Python’s *textstat* library (v3.11; Python Software Foundation, Wilmington, DE, USA) or equivalent R-based packages. The formulas used were as follows:

FRES = 206.835 − (1.015 × WCSS) − (84.6 × WCSYC)

FKGL = (0.39 × SSWC) + (11.8 × WCSYC) − 15.59

where WC = word count, SS = sentence count, WCS = words per sentence, YC = syllable count, and SYC = syllables per word.

To evaluate the quality and reliability of the chatbot responses, two independent, blinded physician reviewers with clinical experience in nephrology used the Ensuring Quality Information for Patients (EQIP) tool and the Modified DISCERN instrument [11,12]. The EQIP score was calculated as a percentage using the following formula:

$$\mathrm{Score}\;=\;\frac{\left(Yes\times 1)+(Partly\times 0.5\right)}{20-Not\;Applicable}\;\times \;100$$

Here, Yes denotes the number of criteria fully met, Partly indicates partial fulfillment, and Not Applicable refers to items excluded from scoring. Scores between 0–25% indicated severe quality issues, 26–50% serious deficiencies, 51–75% good quality with minor issues, and 76–100% well-written content.

The Modified DISCERN score ranged from 1 (very poor quality) to 5 (excellent quality). Any discrepancies between reviewers were resolved by a third evaluator.

As this study did not involve human participants or patient responses, no data regarding patients’ educational level or dialysis status were collected. The question set was designed to reflect commonly encountered concerns during PD counseling rather than characteristics of a specific patient population. The full list of questions, standardized prompts, and anonymized scoring templates are provided in Supplementary File S1 to support transparency and reproducibility.

## 3. Statistical Analysis

All statistical analyses were performed using IBM SPSS Statistics for Windows, Version 20.0 (IBM Corp., Armonk, NY, USA) [IBM Corp. Released 2011]. Categorical variables were expressed as numbers and percentages, whereas continuous variables were summarized as mean and standard deviation and as median and IQR (interquartile range) where appropriate. The normality of distribution for continuous variables was confirmed with the Shapiro–Wilk test. For comparison of three chatbot groups, one-way ANOVA or Kruskal–Wallis test was used depending on whether the statistical hypotheses were fulfilled or not. For normally distributed data, regarding the homogeneity of variances, Bonferroni or Games&Howell tests were used for multiple comparisons of chatbot groups. For nonnormally distributed data, Bonferroni adjusted Mann–Whitney U test was used for multiple comparisons of chatbot groups. The statistical level of significance for all tests was considered to be 0.05.

## 4. Results

Regarding textual characteristics, Gemini Pro 2.5 produced the longest and most detailed responses with higher word, sentence, and syllable counts. For general information questions, the median word count (IQR) was highest for Gemini Pro 2.5 (873 [759–925]), followed by ChatGPT-5 (346 [283–436]) and LLaMA Maverick 4 (352 [212–376]) (*p* < 0.001). Similar trends were observed for sentence and syllable counts, with Gemini outperforming both models (*p* < 0.001). LLaMA Maverick 4 consistently generated the shortest and least elaborate answers, while ChatGPT-5 demonstrated an intermediate performance. Detailed comparisons of word counts, syllable counts, and sentence counts across the three chatbots are shown in Table 2. These findings highlight substantial variation among the models in terms of content richness and elaboration.

**Figure 1:**
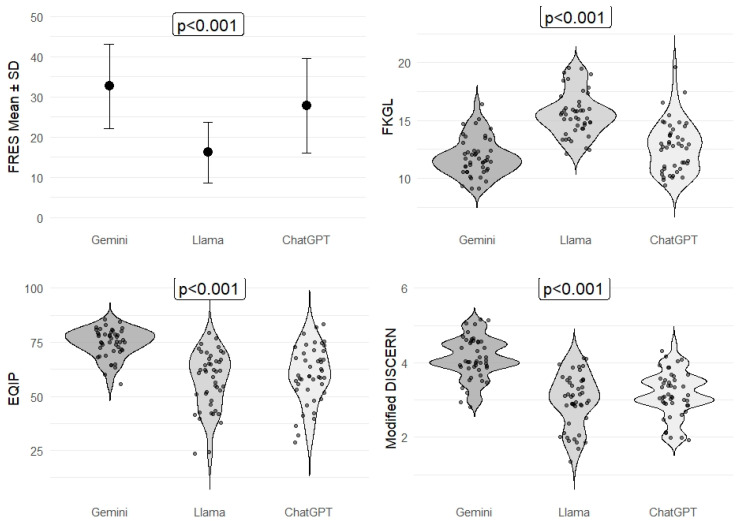
Readability and Quality Metrics of AI Chatbots for Peritoneal Dialysis Education.

Readability analysis using the Flesch Reading Ease Score (FRES) showed a comparable pattern across chatbot models. Gemini achieved the highest FRES values across general information (35.9 ± 5.6), technical/clinical issues (33.8 ± 4.7), and myths/misconceptions (28.3 ± 3.9), all significantly higher than those of LLaMA Maverick 4 (18.0 ± 8.0, 17.6 ± 7.1, and 14.9 ± 6.5, respectively; *p* < 0.001). ChatGPT-5 demonstrated intermediate FRES scores across all categories. Although all responses were classified within the ‘difficult’ readability range (FRES <50), statistically significant differences in FRES values were observed among the models. Detailed readability (FRES, FKGL) and content quality/reliability (EQIP, Modified DISCERN) scores for all subgroups and overall comparisons are presented in Table 3, and summarized in Figure 1.

Assessment of content quality and reliability revealed significant differences among the chatbots. Overall EQIP scores were highest for Gemini Pro 2.5 (median [IQR]: 75.4 [71.3–81.9]), followed by LLaMA Maverick 4 (61.5 [47.2–67.3]) and ChatGPT-5 (59.4 [53.1–70.4]) (*p* < 0.001). Similarly, Modified DISCERN scores were highest for Gemini Pro 2.5 (4.0 [4.0–4.5]), compared with ChatGPT-5 (3.5 [3.0–4.0]) and LLaMA Maverick 4 (3.0 [2.5–3.5]) (*p* < 0.001).

Taken together, the findings demonstrate consistent differences among the evaluated chatbots across textual characteristics, readability, and quality metrics.

## 5. Discussion

This study is one of the first to systematically evaluate the responses of three different AI-based chatbots to frequently asked questions about PD in terms of readability, content quality, and reliability. It is well established that limited patient education, misconceptions, and barriers to accessing accurate information play a significant role in the underutilization of PD worldwide [13,14]. In recent years, AI-based chatbots have gained increasing attention for their potential to improve health literacy and support patient–physician communication [14,15,16]. However, uncertainties regarding the quality, reliability, and clinical applicability of the information they provide remain unresolved.

This study was not designed to validate AI-generated responses against established clinical standards or physician-provided information. Rather, the primary aim was to conduct a comparative evaluation of different large language models under identical, context-free conditions to highlight relative differences in readability, content structure, and quality metrics. Accordingly, comparisons were made among AI chatbots rather than against a clinical gold standard. While such an approach does not establish clinical validity, it provides insight into the variability of AI-generated information and underscores the need for cautious interpretation and further validation studies involving clinician benchmarks.

In this study, Gemini Pro 2.5 showed higher values across textual features (word, sentence, and syllable counts), as well as readability and content quality metrics (FRES, EQIP, and Modified DISCERN), compared with the other evaluated models. Higher word and sentence counts indicate that the responses generated by Gemini were more detailed in structure under the evaluated conditions. In contrast, LLaMA Maverick 4 generated lower values across these metrics, while ChatGPT-5 demonstrated intermediate performance. These findings are consistent with previous reports suggesting that Google-based language models tend to generate longer and more elaborated responses [13].

Several questions included in the evaluation were inherently context-dependent and do not have a single universally correct answer. Accordingly, AI-generated responses should be interpreted as general informational outputs rather than individualized recommendations.

The finding that FRES scores were significantly higher for Gemini across all domains is noteworthy. However, it is important to emphasize that readability scores for all three chatbots remained within the ‘difficult’ range (FRES < 50). Therefore, these differences should be interpreted as relative rather than absolute improvements in readability. Although longer texts are often assumed to be less readable, Gemini-generated responses demonstrated comparatively higher FRES scores despite greater length, suggesting better linguistic organization under identical prompting conditions. In contrast, LLaMA produced both shorter responses and lower readability scores. These differences may reflect variation in language processing capabilities and training data characteristics among models [17].

An important finding of this study is that all evaluated AI chatbots generated responses within the ‘difficult’ readability range (FRES < 50), representing a major barrier to real-world peritoneal dialysis (PD) decision-making. This limitation is particularly relevant for patients newly diagnosed with kidney failure, who often experience high cognitive load and decisional anxiety. Current recommendations for patient education materials generally target a readability level corresponding to approximately the 6th–8th grade. Achieving this threshold may require deliberate linguistic simplification strategies, such as shorter sentences, avoidance of medical jargon or the inclusion of plain-language definitions, structured bullet-point formats, and teach-back prompts to reinforce understanding. While response length was not treated as an independent marker of quality in this study, longer responses are not inherently superior in the context of patient education. Rather, textual length should be interpreted in conjunction with readability indices and content quality metrics, as greater length may reflect more comprehensive coverage only when accompanied by improved clarity and patient-centered presentation.

Beyond technical performance, patient trust is a critical determinant of the real-world utility of AI-based educational tools. Previous patient-centered research has shown that although many patients believe AI may improve healthcare, substantial concerns persist regarding diagnostic errors, data privacy, and increased healthcare costs. In a recent cross-sectional survey of patients, Erul et al. reported that one-third of participants were very uncomfortable with AI-led diagnoses and that concerns about incorrect decisions and data confidentiality were particularly pronounced among individuals with lower educational levels. These findings suggest that the quality, reliability, and readability of AI-generated information are central to patient acceptance and underscore the importance of careful evaluation and clinician oversight when integrating chatbot-based tools into patient education [18].

Higher EQIP and Modified DISCERN scores observed for Gemini suggest comparatively stronger performance in content quality and reliability metrics under the evaluated conditions. LLaMA’s lower scores reflect its weaker performance in medical contexts, consistent with previous literature [13]. These results complement prior studies demonstrating the expanding role of AI and machine learning (ML) algorithms in peritoneal dialysis, including the prediction of peritonitis, technique failure, and cardiovascular complications, highlighting that AI applications extend beyond patient education to encompass risk stratification and individualized patient care [19].

These findings are consistent with recent studies in CKD patient education, which reported overall high accuracy for ChatGPT-5 and other AI models but also noted occasional misleading responses and lack of consistent referencing. Importantly, those studies observed performance improvements with newer AI versions, suggesting that iterative model refinement and alignment with clinical guidelines may further enhance reliability [10].

Our findings are similar to reports from other fields, such as oncology and hepatology, where model-dependent variations in performance have also been documented. For example, prior studies have shown that while ChatGPT-5 often provides accurate information, limitations in contextual appropriateness necessitate caution in clinical applications [16]. Our findings also align with recent evidence showing that AI-generated medical responses can achieve high levels of accuracy and completeness across diverse clinical topics [9]. However, by incorporating readability and patient education metrics, our study expands upon this prior work, highlighting dimensions beyond correctness alone.

ML algorithms have demonstrated strong predictive performance in forecasting PD complications and improving patient outcomes [13,17]. Our study highlights the additional value of AI in the realm of patient education. Integration of AI with remote patient management (RPM) and telemedicine applications has the potential to enhance both patient satisfaction and outcomes, particularly in aging populations [14]. The observed performance differences among chatbots underscore the importance of model selection in such integration efforts.

Furthermore, the incorporation of multi-omics data with AI in diabetic PD patients offers promising opportunities for the development of personalized nutritional and therapeutic strategies [20]. Our findings suggest that selecting the appropriate model will be crucial to ensuring the reliability of such data-intensive applications.

One notable finding of this study was the complete absence of references across all responses generated by the three AI chatbots. In the EQIP assessment, the item ‘Are sources or references provided?’ consistently received negative ratings from all reviewers. Although prompting the models explicitly to provide references can yield citation-like outputs, prior evidence and our own observations indicate that the accuracy of such references including journal name, year, and content validity cannot be guaranteed without manual verification. This limitation underscores a critical barrier to the safe and responsible use of AI chatbots in healthcare education at the current stage of technological development.

One of the strengths of our study is the use of validated tools such as EQIP and Modified DISCERN, with independent, blinded reviewers assessing all responses, thereby minimizing subjective bias. It should also be noted that LLMs are trained on globally diverse datasets and do not learn from real-time user interactions; therefore, geographical variations in usage intensity are unlikely to affect model performance. However, access to certain chatbots, such as ChatGPT-5, remains restricted in some regions due to infrastructural and legal limitations rather than training-related factors, and whether this introduces bias warrants further methodological investigation.

While accuracy, readability, and content quality are essential components of patient education, this study did not assess whether AI-generated information directly altered clinical or patient decision-making. Decision-making outcomes, such as modality choice or treatment adherence, were beyond the scope of the present analysis. Nevertheless, access to clear, reliable, and comprehensible information represents a fundamental prerequisite for informed decision-making.

Patient education in peritoneal dialysis is inherently preference- and lifestyle-sensitive. Factors such as work obligations, caregiving roles, physical appearance, travel flexibility, and home conditions strongly influence both modality choice and treatment adherence. Although patients were not directly involved in generating the FAQ set, the included questions intentionally targeted these real-world concerns. Nevertheless, the consistently low readability scores observed across all evaluated chatbots highlight an important equity issue. Patients with limited health literacy, restricted digital access, disabilities, or language barriers may face additional challenges when relying on AI-generated educational content. These findings underscore the need for clinician-mediated use of AI tools, readability optimization, and future co-design approaches involving patients and caregivers to ensure equitable and accessible PD education.

Although several AI-based chatbots offer free access with basic functionality, more advanced model versions—such as those used in the present study—may require paid subscriptions, and large-scale clinical implementation may involve additional costs related to infrastructure, data governance, and clinician oversight.

Limitations of our study include the evaluation of only three models, restriction to English-language content, and the lack of assessment of clinical outcomes. Although the Modified DISCERN instrument was applied using an overall score, individual sections of the DISCERN framework address different dimensions of information quality and clinical decision-making. Section-specific weighting was not performed in this study and may represent an important consideration for future research focusing on decision support. Although independent, context-free sessions were used to minimize memory effects, large language models may still generate variable responses due to inherent stochasticity. Moreover, because AI models are frequently updated, our findings reflect model performance during a specific timeframe.

## 6. Conclusions

This study provides the first comprehensive and multidimensional evaluation of AI-based chatbots in peritoneal dialysis patient education. It does not aim to recommend any specific AI chatbot for independent patient use. Rather, it highlights measurable differences among large language models when responding to identical PD-related questions, emphasizing the need for cautious interpretation and clinician oversight. Importantly, AI-generated information should not replace professional medical advice and must always be interpreted within appropriate clinical context and safeguards.

## Figures and Tables

**Table 1:** Categories of Questions: General Information, Technical & Clinical Concerns, and Myths & Misconceptions.

No	Question (English)
General Information	
1	What is peritoneal dialysis?
2	What is the difference between peritoneal dialysis and hemodialysis?
3	Which is more effective: peritoneal dialysis or hemodialysis?
4	Who decides if peritoneal dialysis is right for me, and can I switch later?
5	When do I need to start peritoneal dialysis?
6	How does peritoneal dialysis work?
7	How much time per day does peritoneal dialysis take?
8	What are the different types of peritoneal dialysis (CAPD and APD)?
9	What is the difference between CAPD (continuous ambulatory peritoneal dialysis) and APD (automated peritoneal dialysis) in terms of lifestyle?
10	How often do patients need to perform peritoneal dialysis?
11	How do I do peritoneal dialysis?
12	What kind of training is required before starting peritoneal dialysis?
13	Can patients travel or go on vacation while on peritoneal dialysis?
14	Can I continue working while on peritoneal dialysis?
15	Will people notice that I am on peritoneal dialysis because of my appearance?
Technical & Clinical Concerns	
1	Can I do peritoneal dialysis by myself?
2	If I have a disability, can I perform peritoneal dialysis on my own?
3	How does assisted peritoneal dialysis work?
4	Do I have to do peritoneal dialysis every day?
5	Does peritoneal dialysis require a special diet?
6	Can constipation affect my peritoneal dialysis catheter or cause complications?
7	How is fluid overload managed in peritoneal dialysis patients?
8	Will the peritoneal dialysis catheter trigger alarms at the airport?
9	Can I swim while on peritoneal dialysis?
10	Can I have sex while on peritoneal dialysis?
11	Does peritoneal dialysis catheter placement require surgery?
12	What should I expect after catheter insertion of peritoneal dialysis (healing, restrictions, pain)?
13	Can peritoneal dialysis be used in patients with abdominal surgery history?
14	Does long-term peritoneal dialysis damage the peritoneal membrane?
15	What are the most common complications of peritoneal dialysis?
16	How can I recognize peritonitis in peritoneal dialysis?
17	How is peritonitis treated in peritoneal dialysis?
18	How can the risk of infection be minimized in peritoneal dialysis?
19	How should catheter exit site care be performed for peritoneal dialysis patients?
20	What physical activities or sports should I avoid with peritoneal dialysis?
21	What happens if my peritoneal dialysis catheter gets accidentally pulled or injured?
Myths & Misconceptions	
1	Is peritoneal dialysis less effective than hemodialysis?
2	Does peritoneal dialysis make your belly permanently swollen or look abnormal?
3	Is peritoneal dialysis only suitable for poor or rural patients?
4	Does peritoneal dialysis always cause infections?
5	Do patients on peritoneal dialysis gain excessive weight due to fluid absorption?
6	Can people on peritoneal dialysis never go outside or travel freely?
7	Are peritoneal dialysis solutions delivered to only one place?
8	Is peritoneal dialysis not recommended for elderly patients?
9	Does peritoneal dialysis mean I cannot live with pets at home?

**Table 2:** Comparison of Word Counts, Syllables, and Sentences Across Chatbots.

	Word Count	*p*	Syllables Count	*p*	Sentence Count	*p*
General Information						
Gemini	873.0 (759.0–925.0) ^α,β^	<0.001	1588.5 ± 235.2 ^α,β^	<0.001	74.0 (57.0–82.0) ^α,β^	<0.001
Llama	352.0 (212.0–376.0)		667.0 ± 254.2		23.0 (17.0–29.0)	
ChatGPT-5	346.0 (283.0–436.0)		733.2 ± 359.7		29.0 (20.0–38.0)	
Technical & Clinical Concerns						
Gemini	862.4 ± 121.3 ^α,β^	<0.001	1654.0 (1470.0–1845.5) ^α,β^	<0.001	68.9 ± 15.5 ^α,β^	<0.001
Llama	360.1 ± 93.1		751.0 (594.0–920.5)		21.9 ± 7.2 ^β^	
ChatGPT-5	376.9 ± 167.4		694.0 (507.0–951.5)		32.4 ± 15.8	
Myths & Misconceptions						
Gemini	726.3 ± 146.5 ^α,β^	<0.001	1415.8 ± 288.6 ^α,β^	<0.001	55.2 ± 12.2 ^α,β^	<0.001
Llama	321.8 ± 103.7		663.6 ± 212.4		17.2 ± 5.9	
ChatGPT-5	283.4 ± 99.8		560.6 ± 188.8		22.0 ± 8.9	
Total						
Gemini	840.0 (747.5–915.5) ^α,β^	<0.001	1601.0 (1427.5–1757.5) ^α,β^	<0.001	66.0 (56.0–77.5) ^α,β^	<0.001
Llama	352.0 (270.0–413.5)		715.0 (584.5–900.0)		20.0 (17.0–27.0)	
ChatGPT-5	334.0 (261.0–448.5)		671.0 (524.0–867.0)		28.0 (20.0–38.5)	

Data was expressed as mean ± standard deviation or median (IQR). ^α^ *p* < 0.05 compared with Llama, ^β^ *p* < 0.05 compared with ChatGPT-5.

**Table 3:** Comparison of Flesch Reading Ease Scores (FRES) Among Chatbots Across All Subgroups and Overall.

	FRES	*p*	FKGL	*p*	EQIP	*p*	Modified DISCERN	*p*
General Information								
Gemini	35.9 ± 5.6 ^α^	<0.001	11.2 ± 0.8 ^α^	<0.001	70.9 ± 5.9 ^α^	0.013	4.0 (3.5–4.0) ^α^	0.006
Llama	18.0 ± 8.0 ^β^		14.5 ± 1.4 ^β^		57.3 ± 14.7		3.0 (2.0–3.5)	
ChatGPT-5	28.7 ± 11.3		12.3 ± 1.8		60.3 ± 15.3		3.5 (3.0–4.0)	
Technical & Clinical Concerns								
Gemini	32.2 ± 12.3 ^α^	<0.001	11.9 ± 1.9 ^α^	<0.001	78.5 ± 4.3 ^α,β^	<0.001	4.5 (4.0–4.5) ^α,β^	<0.001
Llama	16.6 ± 6.9 ^β^		15.9 ± 1.9 ^β^		61.0 ± 9.3		3.0 (3.0–3.5)	
ChatGPT-5	28.1 ± 12.8		12.9 ± 2.7		61.5 ± 12.2		3.5 (2.8–3.5)	
Myths & Misconceptions								
Gemini	28.0 ± 11.3 ^α^	0.005	12.6 ± 1.7 ^α^	<0.001	74.9 (67.9–77.7) ^α,β^	0.002	4.0 (3.8–4.5) ^α,β^	0.001
Llama	12.1 ± 7.0 ^β^		16.4 ± 1.8 ^β^		51.2 (33.4–61.8)		2.5 (1.8–3.0)	
ChatGPT-5	25.4 ± 10.8		13.0 ± 1.4		59.4 (56.8–62.7)		3.0 (3.0–3.0)	
Total								
Gemini	32.6 ± 10.5 ^α^	<0.001	11.6 (10.6–12.8) ^α^	<0.001	75.4 (71.3–81.9) ^α,β^	<0.001	4.0 (4.0–4.5) ^α,β^	<0.001
Llama	16.2 ± 7.5 ^β^		15.5 (14.3–16.4) ^β^		61.5 (47.2–67.3)		3.0 (2.5–3.5)	
ChatGPT-5	24.2 ± 11.7		12.8 (10.8–14.1)		59.4 (53.1–70.4)		3.0 (3.0–3.5)	

Data was expressed as mean ± standard deviation or median(IQR). ^α^ *p* < 0.05 compared with Llama, ^β^ *p* < 0.05 compared with ChatGPT-5.

## Data Availability

Data supporting the findings of this study are available from the corresponding author upon reasonable request.

## Supplementary Materials

The following supporting information can be downloaded at: https://www.mdpi.com/article/10.3390/jcm15020692/s1. File S1: Question Set, Standardized Prompts, and Evaluation Tools Used in the Study.
