# Supplementary material for: Artificial Intelligence Chatbots in Peritoneal Dialysis Education: A Cross-Sectional Comparative Study of Quality, Readability, and Reliability
Source: J Clin Med. 2026 Jan 15;15(2):692. doi: 10.3390/jcm15020692 (PMC12842389; doi:10.3390/jcm15020692)
Supplement: Supplementary file 1 [file jcm-15-00692-s001.zip › jcm-4092819-supplementary.pdf]

Supplementary File 1

Question Set, Standardized Prompts, and Evaluation Tools Used in the Study

1. Full List of Peritoneal Dialysis–Related Questions

This section provides the complete list of 45 frequently asked questions used to evaluate AI-based chatbots in peritoneal dialysis education. The questions were grouped into three domains: General Information (15 questions), Technical & Clinical Concerns (21 questions), and Myths & Misconceptions (9 questions). All questions were submitted to each chatbot using identical wording.

Questions

General Information

No	Question (English)
1	What is peritoneal dialysis?
2	What is the difference between peritoneal dialysis and hemodialysis?
3	Which is more effective: peritoneal dialysis or hemodialysis?
4	Who decides if peritoneal dialysis is right for me, and can I switch later?
5	When do I need to start peritoneal dialysis?
6	How does peritoneal dialysis work?
7	How much time per day does peritoneal dialysis take?
8	What are the different types of peritoneal dialysis (CAPD and APD)?
9	What is the difference between CAPD (continuous ambulatory peritoneal dialysis) and APD (automated peritoneal dialysis) in terms of lifestyle?
10	How often do patients need to perform peritoneal dialysis?
11	How do I do peritoneal dialysis?
12	What kind of training is required before starting peritoneal dialysis?
13	Can patients travel or go on vacation while on peritoneal dialysis?
14	Can I continue working while on peritoneal dialysis?
15	Will people notice that I am on peritoneal dialysis because of my appearance?

Technical & Clinical Concerns

No	Question (English)
1	Can I do peritoneal dialysis by myself?
2	If I have a disability, can I perform peritoneal dialysis on my own?
3	How does assisted peritoneal dialysis work?
4	Do I have to do peritoneal dialysis every day?
5	Does peritoneal dialysis require a special diet?
6	Can constipation affect my peritoneal dialysis catheter or cause complications?
7	How is fluid overload managed in peritoneal dialysis patients?
8	Will the peritoneal dialysis catheter trigger alarms at the airport?
9	Can I swim while on peritoneal dialysis?
10	Can I have sex while on peritoneal dialysis?
11	Does peritoneal dialysis catheter placement require surgery?
12	What should I expect after catheter insertion of peritoneal dialysis (healing, restrictions, pain)?
13	Can peritoneal dialysis be used in patients with abdominal surgery history?
14	Does long-term peritoneal dialysis damage the peritoneal membrane?
15	What are the most common complications of peritoneal dialysis?
16	How can I recognize peritonitis in peritoneal dialysis?
17	How is peritonitis treated in peritoneal dialysis?
18	How can the risk of infection be minimized in peritoneal dialysis?
19	How should catheter exit site care be performed for peritoneal dialysis patients?
20	What physical activities or sports should I avoid with peritoneal dialysis?
21	What happens if my peritoneal dialysis catheter gets accidentally pulled or injured?

Myths & Misconceptions

No	Question (English)
1	Is peritoneal dialysis less effective than hemodialysis?
2	Does peritoneal dialysis make your belly permanently swollen or look abnormal?
3	Is peritoneal dialysis only suitable for poor or rural patients?
4	Does peritoneal dialysis always cause infections?
5	Do patients on peritoneal dialysis gain excessive weight due to fluid absorption?
6	Can people on peritoneal dialysis never go outside or travel freely?
7	Are peritoneal dialysis solutions delivered to only one place?
8	Is peritoneal dialysis not recommended for elderly patients?
9	Does peritoneal dialysis mean I cannot live with pets at home?

2. Standardized Prompting Strategy

All questions were submitted to each chatbot using identical wording and without additional contextual prompts. Each question was entered in a new, isolated chat session equivalent to an 'incognito' mode, without prior conversational history. No follow-up or sequential prompting was performed. Responses were generated in English and recorded verbatim without post-processing or manual editing.

3. Evaluation Tools

Chatbot responses were independently evaluated by two blinded physician reviewers using the Ensuring Quality Information for Patients (EQIP) tool and the Modified DISCERN instrument. The EQIP tool assesses preparation, content quality, and patient-centeredness of health information, while the Modified DISCERN provides an overall quality rating on a 1–5 scale based on accuracy, balance, and decision-support value.

EQIP and Modified DISCERN Combined Evaluation Form

Section 1: EQIP (Ensuring Quality Information for Patients)

Preparation and Structure

• Is the purpose of the material clearly stated?
☐ Yes ☐ Partly ☐ No ☐ Not Applicable

• Is the target audience clearly defined?
☐ Yes ☐ Partly ☐ No ☐ Not Applicable

• Is the content organized in a clear and consistent manner?
☐ Yes ☐ Partly ☐ No ☐ Not Applicable

• Are key messages clearly emphasized?
☐ Yes ☐ Partly ☐ No ☐ Not Applicable

• Is the language patient-friendly and easy to understand?
☐ Yes ☐ Partly ☐ No ☐ Not Applicable

Content Quality

• Does the information include basic details about the disease or condition?
☐ Yes ☐ Partly ☐ No ☐ Not Applicable

• Are treatment options explained?
☐ Yes ☐ Partly ☐ No ☐ Not Applicable

• Are the purposes of each treatment option clearly described?
☐ Yes ☐ Partly ☐ No ☐ Not Applicable

• Are the advantages of treatment options explained?
☐ Yes ☐ Partly ☐ No ☐ Not Applicable

• Are the disadvantages/risks of treatment options described?
☐ Yes ☐ Partly ☐ No ☐ Not Applicable

• Is it stated what may happen if no treatment is given?
☐ Yes ☐ Partly ☐ No ☐ Not Applicable

• Are alternative treatments discussed?
☐ Yes ☐ Partly ☐ No ☐ Not Applicable

• Is the information presented in a balanced and unbiased manner?
☐ Yes ☐ Partly ☐ No ☐ Not Applicable

• Are sources or references provided?
☐ Yes ☐ Partly ☐ No ☐ Not Applicable

• Is the currency of the information indicated (e.g., date)?
☐ Yes ☐ Partly ☐ No ☐ Not Applicable

Patient-Centeredness and Presentation

• Does the material include information that supports patient decision-making?
☐ Yes ☐ Partly ☐ No ☐ Not Applicable

• Is the information structured to anticipate common patient questions?
☐ Yes ☐ Partly ☐ No ☐ Not Applicable

• If graphics, figures, or tables are included, are they clear and easy to understand?
☐ Yes ☐ Partly ☐ No ☐ Not Applicable

• Is the content culturally sensitive and inclusive?
☐ Yes ☐ Partly ☐ No ☐ Not Applicable

• Does the material direct patients to additional information or support resources?
☐ Yes ☐ Partly ☐ No ☐ Not Applicable

Scoring Method

Each item is scored as follows:
Yes: 1 point
Partly: 0.5 points
No: 0 points
Not Applicable (N/A): excluded from the denominator

EQIP Score = (Yes × 1 + Partly × 0.5) / (Total items − N/A) × 100

Section 2: Modified DISCERN Score

Please provide an overall score from 1 to 5 based on the following criteria (individual criteria are not scored separately):

• Is the information accurate and up to date?

• Are sources clearly stated?

• Are risks and benefits presented in a balanced manner?

• Are alternative options discussed?

• Is the information unbiased and free from commercial influence?

• Does the content support informed decision-making?

Overall Rating (1–5): _________

4. Scoring Framework

For EQIP, each item was scored as Yes (1 point), Partly (0.5 points), or No (0 points). Items marked as Not Applicable were excluded from the denominator. EQIP scores were expressed as percentages. Modified DISCERN was applied as a global score ranging from 1 (very poor) to 5 (excellent). Discrepancies between reviewers were resolved by consensus with a third evaluator.
